# Neurovirulent cytokines increase neuronal excitability in a model of coronavirus-induced neuroinflammation

**DOI:** 10.1186/s40635-023-00557-9

**Published:** 2023-10-14

**Authors:** Salil R. Rajayer, Stephen M. Smith

**Affiliations:** 1 Section of Pulmonary, Critical Care, Allergy, and Sleep Medicine, VA Portland Health Care System, 3710 SW U.S. Veterans Hospital Road, R&D 24, Portland, OR 97239 USA; 2 Department of Medicine, Division of Pulmonary, Allergy and Critical Care Medicine, Oregon Health and Science University, Portland, OR 97239 USA

**Keywords:** SARS-CoV-2, COVID-19, Neuronal excitability, Neuroinflammation, Delirium, Encephalopathy

## Abstract

**Background:**

Neurological manifestations of severe coronavirus infections, including SARS-CoV-2, are wide-ranging and may persist following virus clearance. Detailed understanding of the underlying changes in brain function may facilitate the identification of therapeutic targets. We directly tested how neocortical function is impacted by the specific panel of cytokines that occur in coronavirus brain infection**.** Using the whole-cell patch-clamp technique, we determined how the five cytokines (TNFα, IL-1β, IL-6, IL-12p40 and IL-15 for 22–28-h) at concentrations matched to those elicited by MHV-A59 coronavirus brain infection, affected neuronal function in cultured primary mouse neocortical neurons.

**Results:**

We evaluated how acute cytokine exposure affected neuronal excitability (propensity to fire action potentials), membrane properties, and action potential characteristics, as well as sensitivity to changes in extracellular calcium and magnesium (divalent) concentration. Neurovirulent cytokines increased spontaneous excitability and response to low divalent concentration by depolarizing the resting membrane potential and hyperpolarizing the action potential threshold. Evoked excitability was also enhanced by neurovirulent cytokines at physiological divalent concentrations. At low divalent concentrations, the change in evoked excitability was attenuated. One hour after cytokine removal, spontaneous excitability and hyperpolarization of the action potential threshold normalized but membrane depolarization and attenuated divalent-dependent excitability persisted.

**Conclusions:**

Coronavirus-associated cytokine exposure increases spontaneous excitability in neocortical neurons, and some of the changes persist after cytokine removal.

**Supplementary Information:**

The online version contains supplementary material available at 10.1186/s40635-023-00557-9.

## Background

Serious neuropsychiatric manifestations of SARS-CoV-2 infection include impaired cognition, altered attentiveness, reduced consciousness, seizures, and abnormal movements [1–4]. The persistence of some neuropsychiatric features beyond clearance of the infection underlines the need for new treatments [4, 5]. The coronavirus-mediated modifications of neuronal activity and connectivity that underlie these acute and chronic clinical changes are unknown. Changes at the single neuron level will alter interneuronal communication and thereby modify the computational properties of circuits and higher level function [6]. Consequently, therapeutic target identification requires a more detailed understanding of the underlying pathogenesis [7]. Emerging data indicate that neuronal injury in COVID-19 could arise from either sterile inflammation or direct viral infection of the brain [8–12]. The mouse coronavirus, MHV-A59, was utilized as part of an animal model to safely study the actions of the virulent coronaviruses SARS-CoV-1 and MERS-CoV [13]. MHV-A59 is also similar to SARS-CoV-2, as both viruses possess a spike glycoprotein, concentrate in the olfactory mucosa, cause respiratory diseases including ARDS, and lead to acute encephalitis and neuroinflammation [8, 9, 13, 14].

Neuroinflammation, identified by elevated levels of brain cytokines, occurs in COVID-19 and is associated with acute neurological disturbances, persistent structural changes, and severe disease [15–17]. Moreover, markers of neuronal injury such as neuron-specific enolase and S100B correlate with virulence in COVID-19 [18, 19]. MHV-A59 brain infection elevates five proinflammatory cytokines: TNFα, IL-1β, IL-6, IL-12p40, and IL-15. The cytokine signature differs when coronavirus infects the brain compared to when the virus stays outside the central nervous system [20]. We hypothesized that the specific neurovirulent cytokines (NVC), at concentrations matched to those elicited by MHV-A59 coronavirus infection, would change neuronal function. Our rationale was that determining how neuronal function was altered by NVCs would provide a foundation to later identify druggable targets that may reduce or reverse the neuronal and neurological dysfunction associated with viral neuroinflammation [21]. Neocortical neurons were studied because of the global cortical distribution of inflammation in COVID-19 patients with impaired neurological function [3, 22]. We directly assessed neuronal excitability by studying the frequency of action potentials (APs), the fundamental electrical signal in neurons [23, 24]. A priori it was unclear how NVCs would affect neuronal function overall so we used a range of stimuli to more broadly explore the parameter space of excitability. We examined if NVCs affected the response to a range of current injections as well as reduced extracellular divalent concentration (calcium and magnesium). Decreased extracellular calcium concentration ([Ca^2+^]_o_) increases the propensity of neurons to fire APs while reducing the probability of synaptic transmission [29]. Substantial decreases in extracellular calcium occur in association with physiological stimuli and acute neurological insults [25–28]. While viral encephalitis reduces serum calcium and increases extracellular brain levels of calcium-binding proteins [30, 31], it is unclear if brain [Ca^2+^]_o_ is significantly reduced by coronavirus infection. We report that NVCs depolarized neurons and increased baseline excitability while simultaneously changing neuronal sensitivity to the microenvironment. Cytokine clearance promptly normalized baseline excitability without reversing membrane potential depolarization, but the changes in sensitivity to the microenvironment were more complex. The loss of sensitivity to divalents persisted for evoked activity, while spontaneous response to decreased divalents was substantially attenuated following cytokine clearance. These data indicate that at least two mechanisms underlie the changes in neuronal function following exposure to coronavirus-associated cytokines.

## Methods

### Primary neocortical cultures

All animal procedures were approved by VA Portland Health Care System Institutional Animal Care and Use Committee in accordance with the U.S. Public Health Service Policy on Humane Care and Use of Laboratory Animals and the National Institutes of Health Guide for the Care and Use of Laboratory Animals (IRBNetID: 1,659,311, Protocol 4359-20). Neocortical neurons were isolated from 1- to 2-day-old mice of both sexes from a stable breeding colony of wild-type C57BL/6JX129X1 mice as described previously [32]. Briefly, animals were decapitated following anesthesia with isoflurane and cerebral cortices were removed. Cortices were incubated in trypsin and DNase (5 mg/mL and 0.1 mg/mL for 5 min at 34° C) and dissociated with heat polished pipettes. Dissociated cells were maintained in Minimum Essential Medium with Earle’s balanced salt solution (MEM/EBSS, HyClone Labs, South Logan, UT) plus 5% fetal bovine serum (FBS) on glass coverslips in an incubator (humidified air and 5% CO_2_) at 37° C. Cytosine arabinoside (4 µM) was added 48–72 h after plating to limit glial division. Cells were used after 10–30 days in culture.

### Preparation of neurovirulent cytokines

NVC cytokines were applied at final concentrations described in Table 1 below. These levels were selected to match average values measured in astrocytes infected with MHV-A59 for TNFα, IL-6 and IL-12p40 [20]. The concentrations of IL-1β and IL-15 were estimated by multiplying basal values by the expression level ratios for control and infected cells. NVC stock solution was prepared immediately prior to application and contained all cytokines at 100 or 1000-times the final concentrations listed in Table 1 below combined in water. The individual stock solutions used to prepare the NVC solution were made by dissolving lyophilized cytokines (Peprotech, NJ) in MEM plus 5% FBS or water plus 0.1% BSA at 0.1–0.5 µg/µL and stored at −80 °C in individual aliquots. In control experiments, equivalent solutions minus the cytokines were used to treat the cultures.

**Table 1:** Neurovirulent cytokine concentrations (pg/ml) used

Cytokine	TNF-α	IL-1β	IL-6	IL-12 p40	IL-15
Conc. (pg/ml)	185	400	380	690	1

### Electrophysiological recordings

Adjacent coverslips on the same culture plate were treated with NVC solution (1% or 0.1% v/v) or vehicle control (0.0002% BSA) for 22–28 h after which the coverslips were transferred to a recording chamber and continuously perfused with extracellular Tyrode solution (Ca_1.1_) containing (in mM): 150 NaCl, 4 KCl, 10 HEPES, 10 glucose, 1.1 MgCl_2_, 1.1 CaCl_2_, pH corrected to 7.35 with NaOH. Solutions were applied by gravity from a glass capillary (1.2 mm outer diameter) placed 1–2 mm from the neuron under study. Recordings were made using an amplifier (Heka EPC10, Lambrecht, Pfalz, Germany) and 5–10 MΩ resistance electrodes. Recordings were filtered at 2.9 kHz and acquired by digitizing at 20 kHz. Approximately 5 min after establishing whole-cell configuration and balancing the amplifier circuits, neurons were subjected to current injection protocols as described in individual experiments. Recordings were made in Ca_1.1_ prior to switching over to a similar solution with reduced divalents (Ca_0.2_, 0.2 mM CaCl_2_ and MgCl_2_). The patch electrode contained the following (in mM): 135 K-gluconate, 10 HEPES, 4 MgCl_2_, 0.3 NaGTP, 4 NaATP, 10 phosphocreatine disodium, pH corrected to 7.20 with KOH. All reagents were supplied by Sigma-Aldrich (St. Louis, MO). Voltages were corrected for liquid junction potentials. Experiments were performed at 21–23° C.

### Statistical analysis

Analysis was performed using Igor Pro (Wavemetrics, OR). APs were identified as brief deflections from the resting membrane potential (RMP) that peaked at or above − 20 mV. AP threshold was measured as the point at which dV/dt reached 40 mV/ms (see Supplemental Figure s3A). AP amplitude was defined as the voltage difference between membrane potential and peak. AP half-width was defined as the time between rising and falling phases of the AP, measured at the midpoint between the peak and membrane potential. To standardize the approach and minimize variation, measurements were restricted to the first AP elicited by a 40 pA injection. Only neurons that fired APs in both Ca_1.1_ and Ca_0.2_ were subject to analysis of AP characteristics in order to enable paired analysis. All data values were reported as mean (± SE). Statistical tests were performed using IBM SPSS v28 or GraphPad Prism 9. Data from control and NVC-treated neurons at Ca_1.1_ and Ca_0.2_ were analyzed using two-way repeat measures ANOVA. For groups exhibiting a significant interaction between divalent change and NVC treatment (*P* < 0.05), we performed simple main effects analysis. If there was no interaction, we analyzed the individual group differences (NVC or divalents) independently. Post hoc tests were performed when appropriate by Sidak’s multiple comparison tests. For analyzing contingencies, such as comparing the likelihood of neurons staying electrically silent, we used the Fisher exact test. For comparing two individual groups, we used the Student’s *t*-test.

### Microarray analysis of gene expression in neocortical cultures

Data from microarray analysis used to characterize RNA expression levels of receptors for cytokines in these cultures are available at NCBI GEO (GSE218028) [33] and shown in Additional file 1: Table S1.

## Results

### NVC increases spontaneous excitability

Action potentials are transient membrane depolarizations that propagate along neuronal processes and may evoke synaptic transmission, a major form of interneuronal communication. The propensity of neurons to generate APs (excitability) can be evaluated by measuring AP frequency [23, 24]. APs occur when changes in membrane conductance, usually due to presynaptic release of neurotransmitter, depolarize the membrane potential to threshold. Initially, we tested if NVC incubation changed the propensity to fire spontaneous APs under resting conditions. In neurons at resting membrane potential, activity was low and 13/42 neurons (31%) fired APs spontaneously over a 100-s period. NVC-treated neurons were more excitable, and 18/24 (75%) fired APs over the same time (*P* = 0.0008, Fisher’s exact test).

**Fig. 1:**
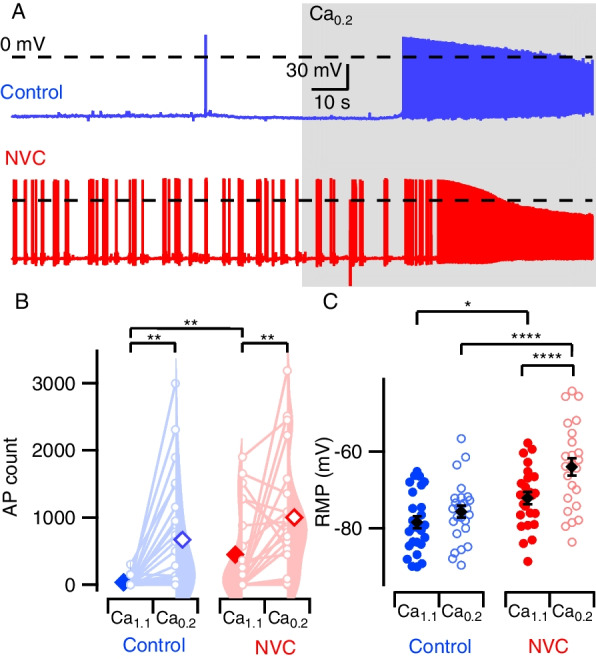
NVC increases spontaneous excitability. **A** Representative voltage tracers of spontaneous APs. Blue represents control and red, NVC. Shaded area indicates Ca_0.2_. Each trace represents 160 s of continuous acquisition. **B** Violin density plots (shaded) showing total AP count at 100 s in Ca_1.1_ and Ca_0.2_. Paired values represented by connected open circles showing change in AP count following divalent switch from Ca_1.1_ to Ca_0.2_. Diamonds indicate mean values, solid Ca_1.1_ and open Ca_0.2_. Mean AP counts in control were 35 ± 15 in Ca_1.1,_ increasing to 670 ± 170 in Ca_0.2_. NVC exposure increased AP counts in Ca_1.1_ to 448 ± 131 and 1002 ± 182 in Ca_0.2._ Two-way repeated measures (RM) ANOVA suggested no interaction between divalent reduction and NVC (*F*(1,45) = 0.099, *P* = 0.753). However, both divalents (*P* < 0.0001) and NVC (*P* = 0.017) independently increased AP counts. Post hoc testing with Sidak multiple comparisons reveals divalent change increased firing in both control (*P* = 0.001) and NVC (*P* = 0.004). NVC increased AP firing at Ca_1.1_ (*P* = 0.002) but not Ca_0.2_ (*P* = 0.190), *N* = 24 (control) and 23 (NVC), respectively. **C** Plot with individual values of RMP. RMP in control was -78.4 ± 1.5 mV in Ca_1.1_ and −75.7 ± 1.5 mV in Ca_0.2._ NVC treatment depolarized neurons to −72.1 ± 1.6 mV in Ca_1.1_ and −64.0 ± 2.3 mV in Ca_0.2_. Solid circles represent Ca_1.1_ and open circles, Ca_0.2_. Two-way RM ANOVA indicates that divalent reduction and NVC treatment interact to depolarize RMP (*F* (1,48) = 6.281, *P* = 0.016). Post hoc testing with Sidak multiple comparisons reveals that NVC depolarizes RMP at both Ca_1.1_ (*P* = 0.025) and Ca_0.2_** (P** < 0.0001)**.** *N* = 26 (control) and 24 (NVC). Solid diamonds with error bars represent mean ± SEM. Control indicated by blue and NVC, red. Statistically significant *P*-values in this figure and all others denoted by schema * < 0.05, ** < 0.01, *** < 0.001 and **** < 0.0001

We next tested how reduced extracellular divalent concentration affected neuronal excitability, because decreases in external calcium change brain activity in physiological and pathophysiological conditions [25–28]. We counted the spontaneously occurring APs acquired over 100 s in physiological solution (Ca_1.1_), and after reducing extracellular divalent concentrations (Ca_0.2_, Fig. 1A). NVC treatment (*P* = 0.017) and reduced divalent concentration (*P* < 0.0001) both substantially increased AP firing with no interaction (Fig. 1B). Our finding that NVC treatment increases the likelihood of AP generation_,_ indicates neuroinflammatory cytokines change the functional properties of neocortical neurons.

Usually, lowered extracellular divalent concentration increases neuronal excitability, in part, by facilitating activation of voltage-gated sodium channels (VGSCs) and depolarizing the membrane potential [32]. NVC treatment substantially depolarized RMP in Ca_1.1_ (Fig. 1C,  *P* = 0.025) and Ca_0.2_ (*P* < 0.0001). The independent variables interacted such that NVC increased the divalent-dependent depolarization (*P* = 0.016). The enhanced sensitivity to divalents was also illustrated by comparing RMP depolarization upon switching to Ca_0.2_ (2.7 ± 1.1 mV and 8.1 ± 1.9 mV for control and NVC-treated neurons, respectively, *P* = 0.019, Additional file 1: Fig. S1A). NVC and Ca_0.2_ depolarized the membrane potential towards the AP threshold, presumably contributing to the observed increase in excitability. This may arise due to shifts in VGSC gating or activation of other membrane conductances [32, 34–36].

### NVC clearance rapidly restores baseline spontaneous activity

**Fig. 2:**
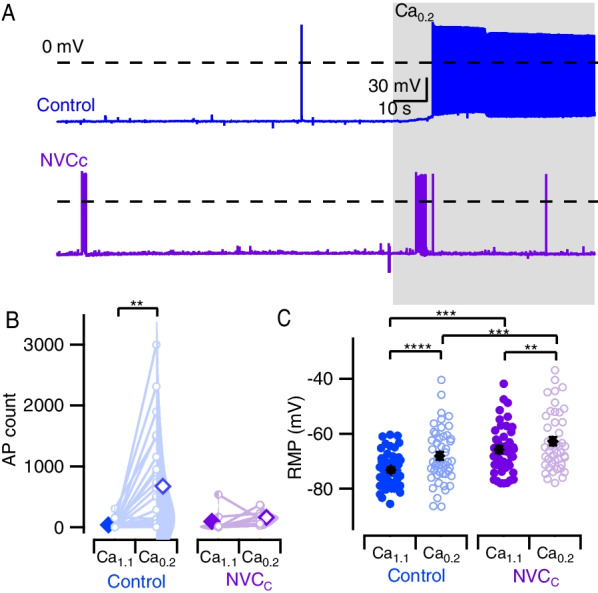
NVC clearance rapidly restores baseline spontaneous activity. **A** Representative voltage tracers for recordings of spontaneous action potentials. Blue tracers represent control and purple NVC clearance (NVC_C_). Shaded area represents application of Ca_0.2_. **B** Violin plots showing distribution of total AP count at 100 s in Ca_1.1_ and Ca_0.2_ in control and NVC_C_, schema similar to Fig. 1B. Mean AP counts in control were 35 ± 15 in Ca_1.1,_ increasing to 670 ± 170 in Ca_0.2_. AP counts following NVC_C_ in Ca_1.1_ and 92.4 ± 66 and 159.1 ± 37.7 in Ca_0.2_. Two-way RM ANOVA did not indicate an interaction between divalent change and NVCc (*P* = 0.063) although decreasing divalents had an overall effect (*P* = 0.024). While change in divalents increased spontaneous AP firing in control (*P* = 0.0013), there was no similar increase noted following NVCc (*P* = 0.99, *N* = 24 and 8, for control and NVCc, respectively). **C** Plots with individual values and mean of RMP. Blue denotes control and purple, NVCc. Individual values represented by solid circles (Ca_1.1_) and open circles (Ca_0.2_). Solid diamonds represent mean values. Mean ± SE for RMP in control; Ca_1.1_ = −73.1 ± 0.9 mV and Ca_0.2_ = −68.0 ± 1.4 mV, in NVCc; Ca_1.1_ = -65.7 ± 1.3 mV and Ca_0.2_ = −62.8 ± 1.5 mV. Two-way RM ANOVA indicates that divalent levels and NVCc had no interaction (*F* (1,93) = 2.578, *P* = 0.112), but both NVCc (*P* = 0.0004) and divalent reduction (*P* < 0.0001) had independent effects. Post hoc testing with Sidak multiple comparisons reveals that RMP was depolarized by NVCc at both Ca_1.1_ (*P* = 0.0002) and Ca_0.2_** (P** = 0.009)**.** *N* = 49 and 46 for control and NVCc, respectively

We evaluated if NVC-mediated changes in neuronal function were affected by the removal of cytokines. The fraction of neurons in which spontaneous APs were recorded ~ 1 h after clearance of NVC (NVCc), was similar to control (*P* > 0.99, 5/15 (33%) of NVCc and 13/42 (31%) of control neurons). Similarly, NVCc reduced the total number of APs generated in Ca_1.1_ in contrast to sustained NVC treatment (Fig. 2B). However, NVCc-treated neurons were surprisingly different to both control and NVC-treated neurons in terms of their sensitivity to external divalent concentration. In NVCc-treated neurons, application of Ca_0.2_ did not change AP count (Fig. 2B; *P* = 0.99), which contrasted with control and NVC-treated neurons (Figs. 1B, 2B). In NVCc-treated neurons, cytokines and reduced divalents both independently depolarized the RMP (Fig. 2C, *P* < 0.0001, *P* = 0.0004) as observed with NVC treatment (Fig. 1C). However, the lack of an interaction between cytokines and reduced divalents represented another difference between NVC- and NVCc-treated neurons (Supplemental Figure s1). The loss of exaggerated depolarization with the switch to low divalent levels in the NVCc-treated neurons, is likely to contribute to the coincident change in divalent-dependent excitability. Overall, while NVC_C_-treated neurons exhibited reduced [Ca^2+^]_o_-dependent excitability, the NVC-mediated changes in RMP persisted.

### NVC attenuates divalent-dependent increase in evoked APs

**Fig. 3:**
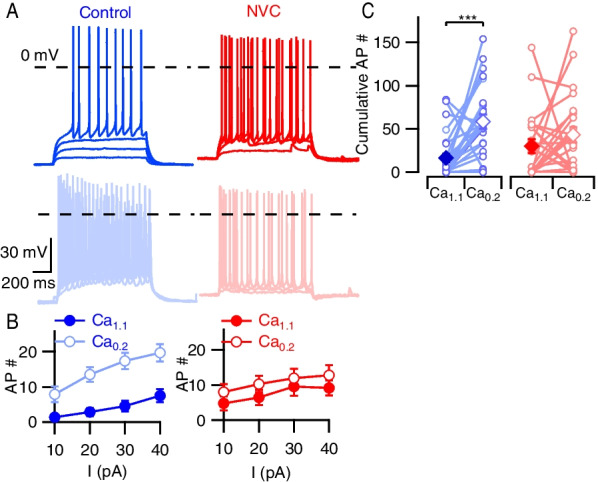
NVC attenuates divalent-dependent increase in evoked APs. **A** Exemplar traces showing AP firing following incremental 1 s current injections of 10–40 pA from RMP. Blue represents control and red NVC. Dark tracers represent Ca_1.1_ and light Ca_0.2_. **B** Graphs showing average AP count in control (blue) and NVC (red), measurements in Ca_1.1_ (solid, filled circles) and Ca_0.2_ (faint, open circles) represented by separate lines. Error bars represent standard error. Mean AP counts in control Ca_1.1_ = 7.5 ± 1.8 and increase to 19.7 ± 2.4 in control Ca_0.2_ at 40 pA, whereas NVC AP counts at the same current injections ranged from 9.2 ± 2.1 at NVC Ca_1.1_ to 12.8 ± 2.9 at NVC Ca_0.2_. **C** Plot showing average cumulative elicited AP count in control (blue) and NVC (red) for each recording. Mean values for Ca_1.1_ represented by solid diamonds and Ca_0.2_ by open diamonds. Mean values for control Ca_1.1_ and Ca_0.2_ were 16.5 ± 5.1 and 58.6 ± 8.0, whereas NVC Ca_1.1_ and Ca_0.2_ were 30.2 ± 7.6 and 43.1 ± 9.0, respectively. Individual values represented by open circles connected by a line showing change in cumulative AP count following switch from Ca_1.1_ to Ca_0.2_. Error bars represent standard error. Comparison by two-way RM ANOVA shows no interaction between divalent concentration and NVC treatment (*F* (1,48) = 3.616, *P* = 0.06) or independent effect of NVC (*P* = 0.91) but decreasing divalents had an effect (*P* = 0.0008). Post hoc analysis with Sidak multiple comparisons shows different effects in control and NVC, with increase in average cumulative AP count in control (*P* = 0.0005) but no effect of NVC (*P* = 0.436). *N* = 26 and 24 for control and NVC groups

In addition to counting spontaneous APs, neuronal excitability can be evaluated by eliciting APs with depolarizing currents [37]. This approach may enhance our ability to detect decreases in excitability by increasing the basal activity. We injected depolarizing currents from RMP and the number of APs increased with current amplitude and Ca_0.2_ as expected for control neurons (Fig. 3A, B, [32]). NVC treatment did not affect neuronal excitability when assessed by the number of APs elicited by current injections (Fig. 3C, *P* = 0.91), in contrast to its action on spontaneous activity (Fig. 1B). Overall, lowering the external divalent concentration increased the AP count (*P* = 0.0008) but this was confined to control neurons (Fig. 3C, *P* = 0.0005). This attenuation of evoked divalent-dependent excitability in NVC-treated neurons contrasted with our observations on spontaneous activity (Fig. 1 and Additional file 1: S1). The membrane potential deflection appeared reduced following current injection in the NVC-treated neurons consistent with a reduced input resistance. However, measurement of the voltage deflection elicited by −20 pA injections revealed the trend towards a lower input resistance was non-significant (*P* = 0.269, Additional file 1: Fig. S2A).

**Fig. 4:**
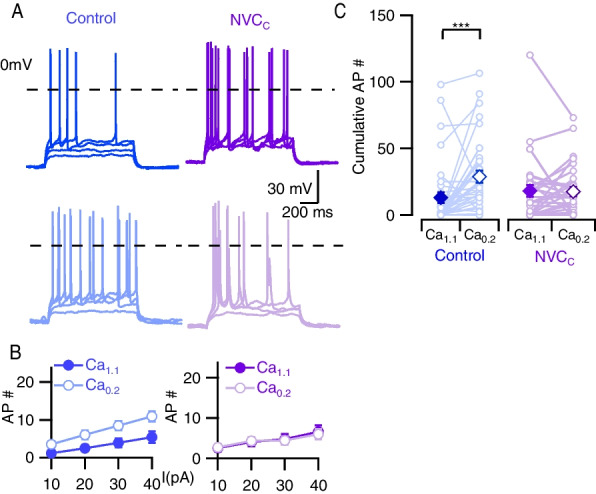
Evoked divalent-dependent excitability remains attenuated after NVC clearance. **A** Exemplar traces showing AP firing following incremental 1-s current injections of 10–40 pA from RMP. Blue represents control and purple NVC clearance (NVCc). Dark tracers represent Ca_1.1_ and light Ca_0.2_. **B** Graphs showing average AP count in control (blue) and NVCc (purple), measurements in Ca_1.1_ (solid, filled circles) and Ca_0.2_ (faint, open circles) represented by separate lines. Error bars represent standard error. 40 pA current injections in control elicited 5.4 ± 1.5 APs in Ca_1.1_ and 10.9 ± 1.3 APs in Ca_0.2_ whereas NVCc elicited 6.6 ± 1.5 APs in Ca_1.1_ and 6.0 ± 1.3 APs in Ca_0.2_. **C** Plot showing average cumulative AP count for each recording. Schema similar to Fig. 3C. Control Ca_1.1_ and Ca_0.2_ = 12.9 ± 4.0 and 28.9 ± 4.4, whereas NVCc Ca_1.1_ and Ca_0.2_ = 18.1 ± 4.4 and 17.61 ± 3.4, respectively. Two-way RM ANOVA showing an interaction between divalent concentration and NVCc (*F* (1,66) = 7.820, *P* = 0.006). Simple main effects analysis showed there was no independent effect of NVCc (*P* = 0.544) but divalent concentration had an independent effect (*P* = 0.010). Post hoc analysis by Sidak multiple comparisons confirmed divalent-dependent increase in excitability was present in control (*P* = 0.003) but not in NVCc (*P* = 0.99). *N* = 37 and 31 for control and NVCc groups

We next examined if cytokine removal affected evoked excitability. NVCc-treated cells were insensitive to the switch from Ca_1.1_ to Ca_0.2_ when assessed by current injection (Fig. 4A–C) similar to NVC-treated neurons (Fig. 3). In addition, both NVC- and NVCc-treated neurons showed trends towards increased basal activity in Ca_1.1._ Taken together, these data indicate that the impact of NVC on excitability depends on the approach used to measure excitability.

### NVC transiently modifies AP threshold

**Fig. 5:**
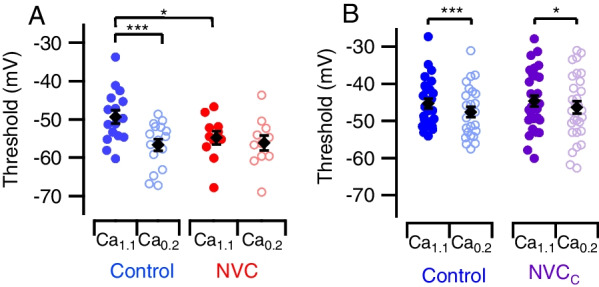
NVC transiently modifies AP threshold. **A** Plot of AP threshold, color schema similar to Fig. 1C. Mean ± SE values for control Ca_1.1_ vs. Ca_0.2_ = −49.3 ± 1.7 mV vs. −56.6 ± 1.5 mV and NVC Ca_1.1_ vs. Ca_0.2_ = −54.8 ± 1.7 mV vs. −56.1 ± 1.9 mV. Two-way RM ANOVA indicates that both divalent levels and NVC interact to hyperpolarize the AP threshold (*F* (1,25) = 5.99, *P* = 0.022). Post hoc testing with Sidak multiple comparisons shows hyperpolarization of AP threshold at Ca_1.1_ between control and NVC (*P* = 0.042), but this effect is absent at Ca_0.2_ (*P* = 0.835). In addition, divalent change hyperpolarized AP threshold in control (*P* < 0.001) but not NVC cells (*P* = 0.481). **B** Plot of AP threshold, color schema similar to Fig. 2C. Mean ± SE values for control Ca_1.1_ vs. Ca_0.2_ = −45.3 ± 1.3 mV vs. −47.6 ± 1.3 mV and NVCc Ca_1.1_ vs. Ca_0.2_ = −44.6 ± 1.5 mV vs. −46.3 ± 1.6 mV. Two-way RM ANOVA indicates that divalent levels and NVCc do not interact to change AP threshold (*F* (1,52) = 0.667, *P* = 0.418), but divalent change had an independent effect (*P* < 0.001). Post hoc testing with Sidak multiple comparisons shows divalent change hyperpolarized AP threshold in both control (*P* = 0.004) and NVCc cells (*P* = 0.041)

There are many mechanisms by which changes in AP shape elicit short- and long-term alterations in neuronal excitability [23, 24, 38]. We tested how AP characteristics were affected by NVC treatment. The effects of NVC treatment and external divalent concentration on AP threshold interacted (Fig. 5A) [32]. As expected, AP threshold was hyperpolarized by Ca_0.2_ in control neurons [32]. In NVC-treated neurons, the AP threshold was relatively hyperpolarized in Ca_1.1_ and unaffected by the switch to Ca_0.2_ (Fig. 5A). Shifts in RMP and threshold will both impact excitability because spike generation is more likely when the gap between RMP and AP threshold decreases which may account for the divalent-dependent evoked excitability in controls (Fig. 3A). However, in NVC-treated neurons there was no significant change in the number of APs following the switch from Ca_1.1_ to Ca_0.2_ (Fig. 3C) despite the relatively hyperpolarized AP threshold and depolarized RMP.

Cytokine-mediated changes to AP threshold were quickly reversed following NVC clearance. AP thresholds (Fig. 5B) were indistinguishable in control and NVCc groups and were similarly hyperpolarized by lowering divalent levels (*P* = 0.004 and 0.041 for control and NVCc, respectively). In NVCc-treated neurons, the absence of divalent-dependent excitability (Fig. 4) despite the divalent sensitivity of AP threshold (Fig. 5B) indicates that AP threshold changes do not explain the cytokine-generated alterations in excitability. NVC did not independently affect AP amplitude (*P* = 0.281) and AP half-maximal width (*P* = 0.796, Additional file 1: Fig. S3) and NVCc had no independent effect on AP amplitude (*P* = 0.082) and AP half-maximal width (*P* = 0.473, Additional file 1: Fig. S4B, C).

## Discussion

Using the MHV-A59 model, originally developed to study the SARS and MERS coronaviruses, we investigated the action of a specific panel of NVCs on neocortical neurons. Our major finding was that day-long exposure to the specific panel of NVCs produced an average 12.8-fold increase in the likelihood of spontaneous action potential generation (Fig. 1B). Despite this large increase in excitability, NVC-treated neurons retained their ability to detect and respond to decreases of the divalent ion concentration in the extracellular environment (Fig. 1B). However, divalent-dependent excitability was lost by NVC-treatment when the APs were elicited by current injection (Fig. 3). We also determined that NVCs depolarized the resting membrane potential, increased the sensitivity of the membrane potential to decreased [Ca^2+^]_o_ and hyperpolarized the AP threshold. Only the effects of NVCs on resting membrane potential and evoked divalent-dependent excitability persisted after cytokine removal (Figs. 2C, 4C). Otherwise, NVC_C_-treated cells and control neurons were indistinguishable except for the unexpected loss of spontaneous divalent-dependent excitability (Fig. 2).

It is not immediately apparent how NVC application caused these many changes in neuronal function and how the changes interacted. The action of low divalents was studied because [Ca^2+^]_o_ decreases by 30–90% during times of high neuronal activity and following acute neurological insult [25–28], and this stimulus modifies neuronal excitability [32, 37, 39]. As mentioned above, we hypothesized that incorporation of [Ca^2+^]_o_ as a parameter would expand our study of the effects of NVC, but we were surprised by their different effects on spontaneous and evoked excitability (Figs. 1, 3). Previously, spontaneous and evoked APs were both used as fairly equivalent measures of excitability [32, 40]. In our recordings, the evoked APs occurred as a result of experimental depolarizations (1 s), whereas spontaneous APs were more likely to result from synaptic inputs. Consequently, the enhanced effect of NVC on spontaneously measured excitability may have arisen if NVCs were acting in part, by increasing excitatory synaptic transmission. Another important finding was the rapid reduction in spontaneous excitability to control levels only one hour after NVC removal (Fig. 2B). On its own, this suggested the impact of NVCs on neuronal function was rapidly reversible consistent with a direct pharmacological effect. However, the associated loss of calcium-dependent excitability (Fig. 2B) indicated some additional sustained effects of NVCs. Persistent effects were also apparent in the experiments evaluating evoked excitability after NVC clearance (Fig. 4B) which were indistinguishable from those where NVCs were not removed. Our evaluation of excitability under a range of conditions, point to two or more mechanisms working together, and that one causes effects that persist for at least an hour after NVC clearance.

NVC depolarized the RMP, hyperpolarized the AP threshold, and increased divalent-dependent depolarization (Fig. 1C, 5A). The changes in RMP and AP threshold both reduced the voltage deflection required to trigger an AP following a depolarizing synaptic input. The heightened sensitivity of the RMP to decreased extracellular divalent concentrations also increased neuronal excitability by further depolarizing the neuron towards the AP threshold. The increase in synaptic activity, hypothesized to explain the pronounced effects on spontaneous excitability (Fig. 1) could arise as a result of changes in RMP, AP threshold, calcium-dependent excitability or represent a distinct action of cytokines. The measured increases in excitability, were all expected to alter the input–output functions of individual neurons, increase the likelihood of AP-evoked synaptic transmission, modify the computational properties of circuits and consequently, behaviors [41–43].

A key finding was the apparent stability of the NVC-induced changes in RMP (Figs. 1C, 2C), contrasting with the reversibility of the changes in AP threshold (Fig. 5), and may point to the mechanisms behind the changes in excitability. Using this approach, the reversal of spontaneous activity in Ca_1.1_ could reflect the change in AP threshold with NVC clearance, whereas the sustained changes in evoked calcium-regulated excitability could depend on the largely intact NVC-mediated changes in RMP. Further complexity is indicated by the complete loss of spontaneous calcium-dependent excitability after NVC clearance which starkly contrasts with the intact calcium-dependent excitability after sustained NVC-treatment. In addition, it may be necessary to invoke downregulation of synaptic activity as a result of cytokine induced homeostatic plasticity [44] to explain the rapid switch from heightened to absent calcium-dependent excitability in the NVC- and NVC_C_-treated neurons.

We have not yet experimentally addressed the mechanisms of action of NVC on neuronal function. The transient effects of NVC on RMP depolarization, AP threshold, and excitability could have arisen in a number of ways. The VGSC is an important candidate because a gating shift could increase channel availability, resulting in an increase in persistent VGSC currents, hyperpolarization of the AP threshold, and an increase in the likelihood of AP generation [32]. This would also explain the enhanced depolarization following the switch to Ca_0.2_ as divalent-dependent depolarization is mainly due to increased VGSC current in these neurons [32]. VGSC regulation by GPCRs and intracellular messengers usually increases VGSC inactivation [45, 46] so the observed NVC actions would require reversal of one of these pathways [47] or a counteracting mechanism [48]. Mechanisms that may contribute to the observed transient effects of NVCs, also include functional changes in one or more other ion channels. For instance, reduced potassium channel activity, including the two-pore leak channels, or increased non-selective channel activity, such as hyperpolarization-activated, cyclic nucleotide gated channel (HCN), at baseline, would depolarize the neurons and increase the likelihood of AP generation. The change in AP threshold might also be attributable to potassium channel closure, while increased divalent sensitivity of the RMP could reflect upregulation of a [Ca^2+^]_o_-sensitive non-selective cation channel such as calcium homeostasis modulator (CALHM1) [36] or the sodium leak channel non-selective protein NALCN [35]. In addition to these post-synaptic mechanisms, enhanced excitatory synaptic transmission could also contribute to the transient effects of NVC by increasing the likelihood of AP generation. The persistent effects of NVC treatment observed after NVC clearance may have arisen from changes in expression levels of membrane proteins that impact the RMP. Candidates include many of the channels listed above as well as the sodium–potassium-ATPase pump. Interest in a potential role for some of these targets is heightened by reports linking their regulation by neuroinflammation. Inhibition of HCN channels has known anti-inflammatory effects [49], while CALHM1 and potassium channels are thought to be activated by inflammation [50]. Future experiments will focus on determining the involvement of these candidate mechanisms in the regulation of excitability by NVCs in order to identify therapeutic targets.

Neuropsychiatric illnesses occur during and following infection with coronaviruses and are important contributors to the morbidity of the COVID-19 pandemic. Inflammation of the brain and the associated encephalopathy may occur early in the disease course, especially in the setting of severe illness [1–3], and present as delirium. Using the MHV-A59 model, originally developed to study severe coronavirus infections, we determined that the viral cytokine signature substantially changed neuronal function. If we were to speculate about the clinical implications of this study, then the changes in neuronal function during (NVC) cytokine application could represent the changes underlying acute illnesses such as delirium or encephalopathy. Likewise, the changes observed in the NVC_C_-treated neurons might explain post-viral illnesses such as long-COVID. Other viral illnesses have also been associated with neuroinflammation, clinical seizures, and chronic neurodegeneration [51, 52]. While there is some overlap in the types of cytokine that are elevated in the various viral encephalitides, it is clear that a distinct group of cytokines has not been identified as responsible for the neuropsychiatric manifestations of all types of viral illness [52, 53]. Individually cytokines have a multiplicity of actions on neuronal function [54, 55], and it is unclear if the cytokine signatures of other viruses would alter neuronal function in the same ways as the NVCs we utilized here. However, the increased neuronal excitability we observed with sustained cytokine exposure, could feasibly predispose to seizures, a common clinical manifestation of encephalitis.

Intrinsic neuronal function and synaptic transmission in the primary neocortical culture share many properties with those observed in the acute brain slice [24, 56] supporting our use of this preparation here. Moreover, the neocortical culture expresses receptors to the NVC panel (Table 1) and its use facilitated the direct and safe testing of how neuronal function was affected by prolonged exposure to the cytokine signature of a coronavirus CNS infection [20]. However, an in vivo model remains an important next step to directly study the pathogenesis of SARS-CoV-2-associated delirium and encephalopathy as it would facilitate the linking of changes in neuronal function with altered behavior. Such a model would also enable studies to determine if the effects of NVCs changed with neurodevelopment. Addressing these limitations, would better position us to identify and test plausible druggable targets to reduce the dysfunction associated with coronavirus neuroinflammation. Another question raised by the study is whether similar changes in neuronal excitability occur with other types of infection? The answer will help determine if the mechanisms underlying neuropsychiatric manifestations of other infections overlap. Finally, experiments extending the duration of exposure with NVC are required to begin understanding the consequences of prolonged neuroinflammation arising from SARS-CoV-2 infection.

## Conclusions

In conclusion, we have described the changes in neuronal function that occur following exposure to the cytokine signature of a serious central coronavirus infection. This dysfunction was partially reversible and included a substantial increase in excitability, altered sensitivity to changes in the extracellular microenvironment, and depolarization of the resting membrane potential.

### Supplementary Information

**Additional file 1: Figure S1.** NVC mediated increase in divalent-dependent depolarization is reversible following NVC clearance. **Figure S2.** Input Resistance of neurons is not altered by NVC or NVC clearance. **Figure S3.** AP Amplitude and AP Half-maximal width are sensitive to divalent change but not NVC. **Figure S4.** AP Amplitude and AP Half-maximal width are unchanged following NVC clearance. **Table S1.** Microarray analysis of cell culture characteristics and cytokine receptors.

## Data Availability

The datasets used and/or analyzed during the current study are available from the corresponding author on reasonable request.
