# Supplementary material for: Neurovirulent cytokines increase neuronal excitability in a model of coronavirus-induced neuroinflammation
Source: Intensive Care Med Exp. 2023 Oct 14;11:71. doi: 10.1186/s40635-023-00557-9 (PMC10575822; doi:10.1186/s40635-023-00557-9)
Supplement: Supplementary file 1 — Additional file 1: Figure S1. NVC mediated increase in divalent-dependent depolarization is reversible following NVC clearance. Figure S2. Input Resistance of neurons is not altered by NVC or NVC clearance. Figure S3. AP Amplitude and AP Half-maximal width are sensitive to divalent change but not NVC. Figure S4. AP Amplitude and AP Half-maximal width are unchanged following NVC clearance. Table S1. Microarray analysis of cell culture characteristics and cytokine receptors. [file 40635_2023_557_MOESM1_ESM.docx]

Online Data Supplement

**Neurovirulent cytokines increase neuronal excitability in a model of coronavirus-induced neuroinflammation**

Salil R. Rajayer, M.B.,B.S, M.C.R and Stephen M. Smith, M.B.,B.S. PhD

**Table of Contents**

1. Supplemental Figure S1: NVC mediated increase in divalent-dependent depolarization is reversible following NVC clearance
2. Supplemental Figure S2: Input Resistance of neurons is not altered by NVC or NVC clearance
3. Supplemental Figure S3: AP Amplitude and AP Half-maximal width are sensitive to divalent change but not NVC
4. Supplemental Figure S4: AP Amplitude and AP Half-maximal width are unchanged following NVC clearance
5. Table S1. Microarray analysis of cell culture characteristics and cytokine receptors


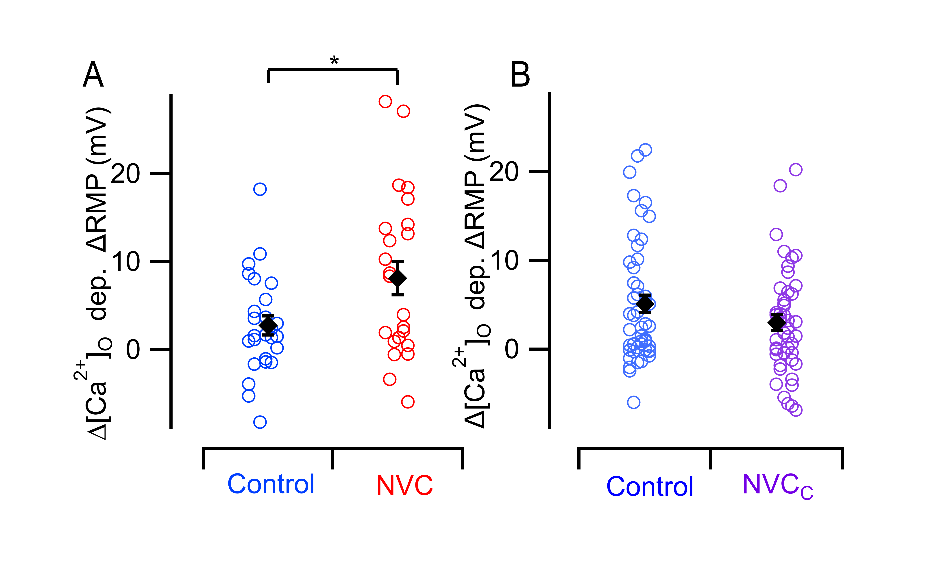


Supplemental Figure S1: **NVC mediated increase in divalent-dependent depolarization is reversible following NVC clearance**

**A.** Plot of individual (open circles) and mean (solid diamonds) values of the magnitude of RMP depolarization following a change in divalent levels in control (blue, mean ± SE = 2.74 ± 1.1 mV) and NVC (red, mean ± SE = 8.11 ± 1.9 mV). Error bars represent standard error. Data was compared by student t-test (P= 0.019). N=26 (control) and 24 (NVC) **B.**  Plot of individual (open circles) and mean (filled diamonds) values showing no significant difference in the magnitude of RMP depolarization following change in divalent levels in control (blue, 5.1 ± 1.0 mV) and NVCc (purple, 3.0 ± 0.9 mV). Data was compared by student t-test (P= 0.110). N= 49 and 46 for control and NVCc respectively.


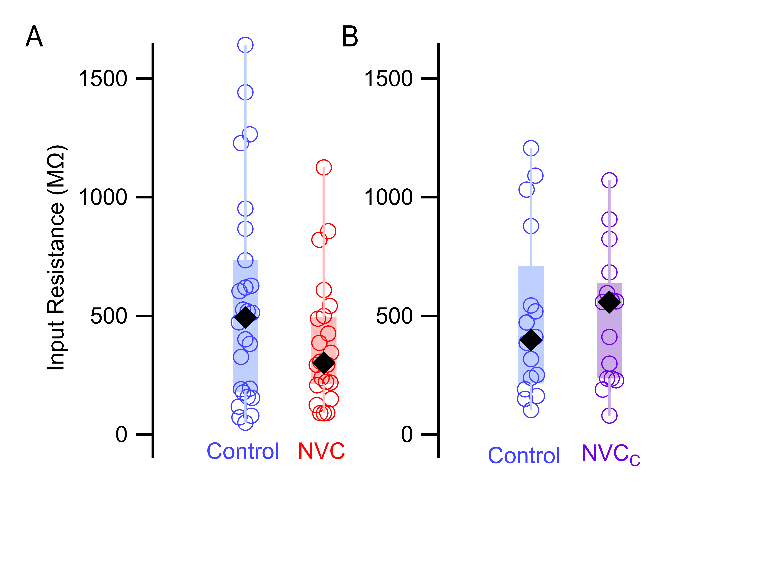


Supplemental Figure S2: **Input resistance of neurons is not altered by NVC or NVC clearance**

**A.** Box plot of individual (open circles) and median (solid diamonds) of input resistance measured as steady state deflection following -20 pA current injection for 1 s in control (blue, median = 493.2 MΩ ) and NVC (red, median = 302 MΩ). Shaded bars represent interquartile ranges. Data compared by Mann-Whitney test showing no significant difference between the two groups (P= 0.269). N=26 (control) and 23 (NVC) **B.** Box plot with schema similar to Figure S2A showing input resistance values in control (blue, 398.7 MΩ) and NVCc (purple, 559.7 MΩ). Data compared by Mann-Whitney test showing no significant difference between the two groups (P= 0.749). N= 16 and 15 for control and NVCc respectively.


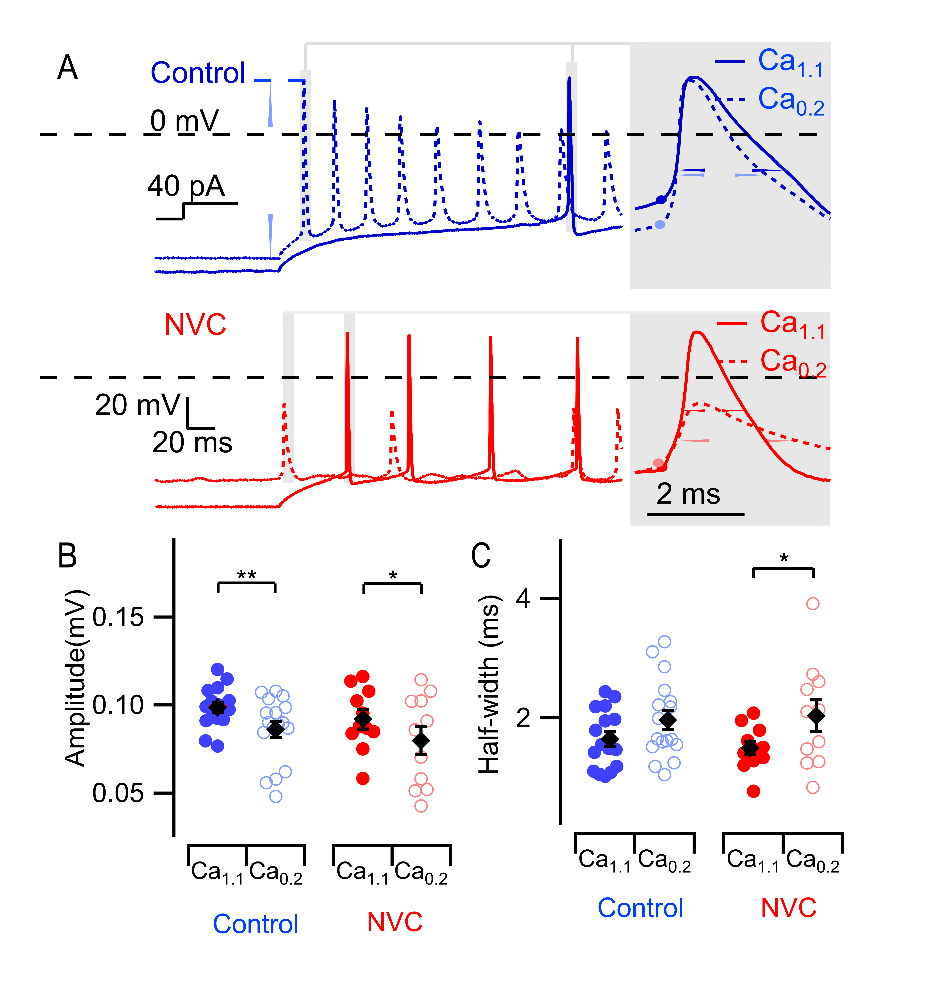


Supplemental Figure S3. **AP Amplitude and AP Half-maximal width are sensitive to divalent change but not NVC**

**A.** Exemplar voltage tracers for train of APs following a 40 pA current injection. Blue represents control and red, NVC. Solid lines represent Ca_1.1_ and interrupted lines, Ca_0.2_. Analysis of the first AP (shaded and magnified in inset) with the following measurements: AP threshold (solid circles), AP half-maximal width (horizontal arrowheads), and AP amplitude (vertical arrowheads). **B.** Plot of AP amplitude. Mean ± SE values for control Ca_1.1_ vs. Ca_0.2_ = 98.7 ± 2.9 mV vs. 86.1 ± 4.9 mV and NVC Ca_1.1_ vs. Ca_0.2_ = 92.0 ± 5.2 mV vs. 79.8 ± 7.8 mV. Two-way RM ANOVA demonstrates no interaction between divalent levels and NVC (F (1,25)= 0.094, P=0.762) or direct effect of NVC (P=0.281). Post-hoc testing with Sidak multiple comparisons shows decreased AP amplitude with divalent change in both control and NVC cells (P=0.005 & 0.044 respectively). **C.** Plot of AP half-maximal width. Mean ± SE values for control Ca_1.1_ vs. Ca_0.2_ = 1.6 ± 0.1 ms vs. 1.9 ± 0.1 ms and NVC Ca_1.1_ vs. Ca_0.2_ = 1.4 ± 0.1 mV vs. 2.0 ± 2.6 mV. Two-way RM ANOVA demonstrates no interaction between divalent levels and NVC (F (1,25)= 0.418, P=0.524) or direct effect of NVC (P=0.796). Post-hoc testing with Sidak multiple comparisons is significant for increased AP half-maximal width with divalent change in NVC but only a trend in control (P=0.028 & 0.083 respectively). N= 16 & 11, control & NVC respectively.


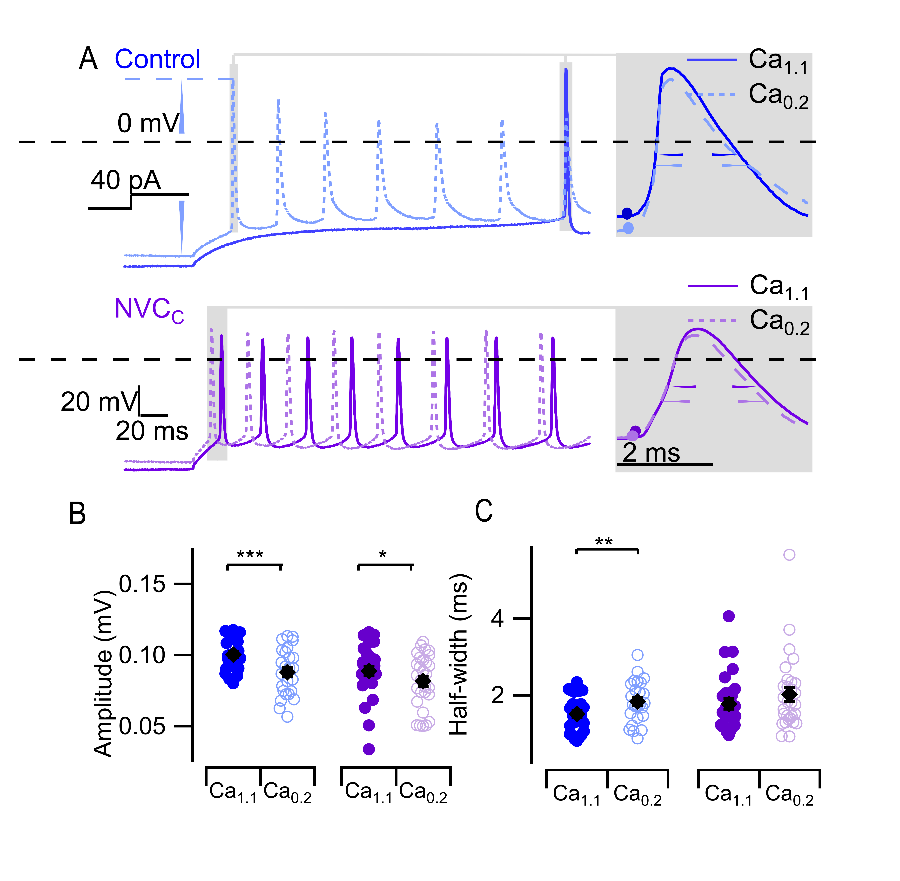


Supplemental Figure S4. **AP Amplitude and AP Half-maximal width are unchanged following NVC clearance**

**A.** Exemplar voltage tracers for train of APs following a 40 pA current injection. Blue indicates control and purple NVCc, solid lines denote Ca_1.1_ and interrupted lines Ca_0.2._ Analysis of the first AP (shaded and magnified in inset) with following measurements: AP threshold (solid circles), AP half-maximal width (horizontal arrowheads) and AP amplitude (vertical arrowheads). **B.** Plot of AP amplitude, Mean ± SE values for control Ca_1.1_ vs. Ca_0.2_ = 100.3 ± 2.3 mV vs. 88.0 ± 3.3 mV and NVCc Ca_1.1_ vs. Ca_0.2_ = 88.8 ± 3.3 mV vs. 81.6 ± 3.5 mV. Two-way RM ANOVA demonstrates no interaction between divalent levels and NVCc (F (1,52)= 3.285, P=0.076) or overall direct effect of NVCc (P=0.082). However divalent change had a significant overall effect (P<0.001). Post-hoc testing with Sidak multiple comparisons shows NVC decreased AP amplitude with divalent change in both control and NVCc cells (P<0.001 & 0.021 respectively). **C.** Plot of AP half-maximal width, Mean ± SE values for control Ca_1.1_ vs. Ca_0.2_ = 1.5 ± 0.0 ms vs. 1.8 ± 0.1 ms and NVCc Ca_1.1_ vs. Ca_0.2_ = 1.8 ± 0.1 ms vs. 2.0 ± 0.1 ms. Two-way RM ANOVA demonstrates no interaction between divalent levels and NVCc (F (1,52)= 1.353, P=0.250) or direct effect of NVCc (P=0.473). Post-hoc testing with Sidak multiple comparisons is significant for increased AP half-maximal width with divalent change in control but only a trend in NVCc (P=0.002 & 0.064 respectively). N= 25 & 29, control & NVCc respectively.

**Table S1: Microarray analysis of cell culture characteristics and cytokine receptors**

| Marker | Gene Name | Gene Symbol | Avg. Expression Level (Log 2) |
| --- | --- | --- | --- |
| Neuron | Synaptophysin | Syp | 13.01 |
| Excitatory Neuron | Calcium/calmodulin-dependent protein kinase II α | Camk2a | 11.66 |
| Excitatory Neuron | Ionotropic glutamate receptor AMPA 2 | Gria2 | 13.43 |
| Inhibitory Neuron | Glutamate decarboxylase 1 | Gad1 | 7.63 |
| Inhibitory Neuron | GABA vesicular transporter | Slc32a1 | 6.31 |
| Microglia | Ionized calcium-binding adapter molecule 1 | Iba-1 | 9.23 |
| Microglia | Transmembrane protein 119 | Tmem119 | 7.08 |
| Astrocyte | Glial-fibrillary acidic protein | Gfap | 16.97 |
| Cytokine Receptors |  |  |  |
| TNF-α | TNF-α receptor 1A | Tnfrsf1a | 12.98 |
| TNF-α | TNF-α receptor 1B | Tnfrsf1b | 6.22 |
| Interleukin-1β | IL-1β receptor | Il1r1 | 9.94 |
| Interleukin-6 | IL-6 receptor α | Il6ra | 7.29 |
| Interleukin-12 | IL-12b1 receptor | Il12rb1 | 2.60 |
| Interleukin-12 | IL-12b2 receptor | Il12rb2 | 4.69 |
| Interleukin-15 | IL-15 receptor | Il15ra | 7.03 |
